# Will Snake Poison Kill a Snake?

**Published:** 1871-06

**Authors:** 


### Will Snake Poison Kill a Snake?

Dr. Fayrer, in India, has been experimenting to correct the popular error that a snake cannot kill a snake. He took a young and very lively cobra, fourteen inches long, and which was bitten in the muscular part of the body by a krait forty-eight inches long. The krait had not bitten for some days before. From a detailed report by Dr. Fayrer, it appears that the cobra was bitten at 12:40 P. M. At 1 P. M. it was very sluggish, at 1:3 P. M. so sluggish that it moved with difficulty, could easily be handled, and made no effort at resistence. At 1:20 it was apparently dying, and its movements were scarcely perceptible, and at 1:22 it died, thirty-two minutes after the attack. Dr. Fayrer has found that the water-snakes of India are deadly poisonous. In the Bay of Bengal they swarm, and it is noted as ominous that lately it was proposed to erect a sea-bathing establishment for Calcutta at Barwar, under the assurance that there were no sharks. It is remarked that sharks need not be noticed when a bather may have deadly water-snakes swimming after him.—*Med. and Surg. Reporter.*

